# Study of Clinicopathological Profile of Sporadic Cases of Colorectal Cancer

**DOI:** 10.5005/jp-journals-10018-1185

**Published:** 2016-12-01

**Authors:** Madhusudan Saha, Bimal C Shil, Shasanka K Saha, Ranjit K Banik, Irin Perveen, MK Sur Chowdhury, ASM Nazmul Islam, ANM Saifullah

**Affiliations:** 1 Department of Gastroenterology, North East Medical College, Sylhet, Bangladesh; 2 Department of Gastroenterology, Sir Salimullah Medical College, Dhaka, Bangladesh; 3 Department of Gastroenterology, Enam Medical College and Hospital, Savar, Dhaka, Bangladesh; 4 Department of Gastroenterology, MAG Osmani Medical College, Sylhet, Bangladesh

**Keywords:** Carcinoma of colon, Profile of patient of ca-colon, Sporadic cases of colorectal cancer.

## Abstract

**Objectives:**

Colorectal cancer (CRC) is the third leading cause of cancer-related deaths in the world. This study was carried out to see the epidemiological and clinicopathological profile of sporadic cases of CRC in Bangladesh.

**Materials and methods:**

The patients diagnosed to have colorectal carcinoma in two private medical centers of Bangladesh from January 2012 to December 2014 were enrolled in this study. Demographic data, clinical presentations, site of lesions, and histological types were analyzed.

**Results:**

Total 158 patients were included in this study. The mean age was 50.77 years and male to female ratio was 1.55:1. Rectal bleeding was the commonest symptom irrespective of age and sex followed by abdominal pain (33, 20.9%), weight loss (29, 18.3%), abdominal mass (26, 16.4%), and altered bowel habit (22, 13.9%). Common histological type was adenocarcinoma (156, 98.7%).

**Conclusion:**

Colorectal cancer commonly affects males >40 years of age. Most common site of involvement is rectum, which is followed by left colon.

**How to cite this article:**

Saha M, Shil BC, Saha SK, Banik RK, Perveen I, Chowdhury MKS, Nazmul Islam ASM, Saifullah ANM. Study of Clinicopathological Profile of Sporadic Cases of Colorectal Cancer. Euroasian J Hepato-Gastroenterol 2016;6(2):134-136.

## INTRODUCTION

Worldwide, colorectal cancer (CRC) is the third most common cancer and the third leading cause of cancer-related death in both males and females.^1,2^ Globally, the highest incidence of CRC rates are in North America, Australia, and Europe, and the lowest rates in Africa and Asia.^3,4^

Incidence of CRC worldwide is 6.5 and 7.7/100,000 females and males respectively. In developed countries, incidence varies from 50 to 60/100,000 population.^3^ It is a rare diagnosis before the age of 40 years, with the incidence beginning to increase significantly between the age of 40 and 50 years and age-specific incidence rates increasing in succeeding decades thereafter.^5^ It has been seen that 90% of new cases are diagnosed in patients over 50 years of age,^6^ but now incidence in younger population is increasing and they present in a more advanced stage.^7^ Recent literature also suggests that there is gradual shift of colon cancer toward right side of colon.^8^ It is also reported that right-sided colon cancer is more in females and rectal cancer is commoner in male.^9^ But in our country, no data regarding incidence or pathological pattern are available. With this background, this study was designed to see the demographic as well as clinicopathological profile of patients with colorectal carcinoma.

## MATERIALS AND METHODS

Consecutive patients diagnosed as cases of CRC in the Department of Gastroenterology of two private medical centers, one in Dhaka and the other in north east part of Bangladesh, were included. Those who did not agree to take part in the study were excluded. The patients were interviewed, and demographic data, clinical and pathological reports were recorded in preformed questionnaire. Age, sex, occupation, family history of cancer (CRC and others), site of lesion, and histopathological types were recorded. Splenic flexure was taken as demarcating point of proximal and distal lesions.

Data were analyzed using Statistical Package for the Social Sciences 16 version. Chi-square test was performed to see significance, and p-value <0.05 was taken as significant.

## RESULTS

A total of 158 patients with CRC were enrolled in this study. Age of the patients varied from 17 to 90 years, with mean 50.77 years. Ninety-six (60.75%) patients were male and 62 (29.25%) were female (Table 1).

**Table 1:** **Table 1:** Demographic data of CRC

*Gender*		*Age ≤ 40 years*		*Age > 40 years*		*Total*	
Male		31 (32.29%)		65 (67.7%)		96 (60.75%)	
Female		22 (35.48%)		40 (64.51%)		62 (39.24%)	
Age stratification		Male		Female		Total	
17–30		14 (56%)		11 (44%)		25 (15.82%)	
31–40		17 (60.71%)		11 (39.29%)		28 (17.72%)	
41–50		19 (67.85%)		9 (32.14%)		28 (17.72%)	
51–60		16 (57.14%)		12 (42.85%)		28 (17.72%)	
>60		30 (61.22%)		19 (38.77%)		49 (31.01%)	
Residence		Male		Female		Total	
Rural		69 (58.47%)		49 (41.52%)		118 (74.68%)	
Urban		27 (67.5%)		13 (32.5%)		40 (25.31%)	

Rectal carcinoma was found in 97 patients, with age varying from 17 to 90 years (mean 48.422 and standard deviation 18.42). Among them, 68 were smokers (p = 0.000) and 82 were betel nut chewers (p = 0.006). Only two patients were alcoholic. Three patients had family history of CRC. Of them, 118 were from rural areas, while rest were from urban areas (p = 0.353) (Table 1).

Main presenting symptoms were altered bowel habit in 28 (17.72%), anemia in 15 (9.49%), melena in 5 (3.16%), bleeding per rectum in 87 (55.06%), abdominal pain with or without features of intestinal obstruction in 33 (20.88%), abdominal mass in 26 (16.45%), and weight loss in 29 (18.35%). Features of obstruction (22, 13.92%) and anemia (11, 6.96%) were the predominant presenting features in proximal lesions, while bleeding per rectum (87, 55.06%), altered bowel habit (22, 13.92%), and pain with obstruction (15, 9.49%) were in distal lesions (Table 2). Commonest site was rectum followed by sigmoid and ascending colon.

**Table 2:** **Table 2:** Comparison of proximal and distal lesions

*Sex*		*Proximal*		*Distal*		*p-value*		*Odds ratio*		*95% confidence interval*	
Male		31		65		0.165		2.278		0.730–7.105	
Female		13		49		0.139					
Clinical presentation											
Bleeding per rectum		1 (1.14%)		86 (98.85%)		0.000		0.002		0.000–0.028	
Pain and/or obstruction		18 (54.54%)		15 (45.45%)		0.108		0.208		0.031–1.413	
Altered bowel habit		6 (21.42%)		22 (78.57%)		0.003		0.060		0.009–0.383	
Mass		16 (61.53%)		10 (38.46%)		0.481		0.579		0.127–2.644	

In this series, 105 patients were in above 40 years age group, while remaining 53 were up to 40 years of age. Proximal colon was affected in 44 patients, while distal colon lesion involvement was in 114 patients. Among them, rectum was affected mostly (97 cases), where the difference was statistically significant (p = 0.00) (Table 3). Overall, rectum was the most common site of lesion.

**Table 3:** **Table 3:** Anatomic distribution of lesion at colonoscopy

Site		Age ≤ 40 years		Age > 40 years		Total	
Rectum + anal canal		41 (42.26%)		56 (57.73%)		97 (36.07%)	
Sigmoid colon		6 (50%)		6 (50%)		12 (7.59%)	
Descending colon		1 (20%)		4 (80%)		5 (3.16%)	
Transverse colon		1 (10%)		9 (90%)		10 (6.32%)	
Ascending colon		2 (8%)		23 (92%)		25 (15.82%)	
Cecum		1 (12.5%)		7 (87.5%)		8 (5.06%)	

All but two were adenocarcinoma histopathologically and remaining two involving anal canal were squamous cell carcinoma.

## DISCUSSION

Colorectal cancer is a major cause of morbidity and mortality throughout the world with large geographical variation.^10^ Though CRC is a disease of old age, nowadays, more younger patients are affected.^11^ In this study, 53 (33.6%) were within 40 years age group, which is higher than that reported from the Western world. This can be explained due to differences in population structures and in life expectancies. Mean age of CRC in our series is 50.77 years, which is earlier in comparison to non-African-Americans in the United States (70.5 years).^12^ Reports from Japan and Korea^13,14^ suggest that incidence of CRC is increasing in Asia. It may be due to change in food habits and lifestyle.

The most common location of CRC in this series is distal to splenic flexure. It is consistent with reports from Malaysia, Islamic Republic of Iran, Japan, Africa, India, and Egypt.^15-20^ However, Western reports^21^ and few reports from Korea^22^ and Japan^17^ show that there is a shift of tumor location to proximal part of colon. In our study, sparing rectal carcinoma, colon carcinoma was more on proximal colon (44 *vs* 34).

Male and female ratio was found to be 1.7:1 in our series (older group 1.62:1, younger group 1.4:1). Overall, CRC incidence and mortality rates are about 35 to 40% higher in men than in women. The reason for this is not completely understood, but likely reflex complex interactions between gender-related differences, exposure to hormones, and risk factors^23^ may be involved.

Symptoms and signs at presentation are different for proximal and distal CRC. Bleeding per rectum and features of obstruction are highly suggestive of distal CRC, while abdominal pain and obstruction, anorexia, anemia, and mass are suggestive of proximal CRC. These are consistent with our series.^24^ In our series, smoking and tobacco use has been found to be a statistically significant associated factor for CRC.
